# Rising to the challenge of defining and operationalising multimorbidity in a UK hospital setting: the ADMISSION research collaborative

**DOI:** 10.1007/s41999-024-00953-8

**Published:** 2024-03-06

**Authors:** Rachel Cooper, Jonathan G. Bunn, Sarah J. Richardson, Susan J. Hillman, Avan A. Sayer, Miles D. Witham, the ADMISSION research collaborative

**Affiliations:** 1 AGE Research Group, Translational and Clinical Research Institute, Faculty of Medical Sciences, Newcastle University, Newcastle upon Tyne, NE4 5PL UK; 2 NIHR Newcastle Biomedical Research Centre, Newcastle Upon Tyne Hospitals NHS Foundation Trust, Cumbria, Northumberland, Tyne and Wear NHS Foundation Trust and Newcastle University, Newcastle upon Tyne, UK

**Keywords:** Multimorbidity, Hospitals, Methods, Definitions, Operationalisation

## Abstract

**Purpose:**

Greater transparency and consistency when defining multimorbidity in different settings is needed. We aimed to: (1) adapt published principles that can guide the selection of long-term conditions for inclusion in research studies of multimorbidity in hospitals; (2) apply these principles and identify a list of long-term conditions; (3) operationalise this list by mapping it to International Classification of Diseases 10th revision (ICD-10) codes.

**Methods:**

Review by independent assessors and ratification by an interdisciplinary programme management group.

**Results:**

Agreement was reached that when defining multimorbidity in hospitals for research purposes all conditions must meet the following four criteria: (1) medical diagnosis; (2) typically present for ≥ 12 months; (3) at least one of currently active; permanent in effect; requiring current treatment, care or therapy; requiring surveillance; remitting-relapsing and requiring ongoing treatment or care, and; (4) lead to at least one of: significantly increased risk of death; significantly reduced quality of life; frailty or physical disability; significantly worsened mental health; significantly increased treatment burden (indicated by an increased risk of hospital admission or increased length of hospital stay). Application of these principles to two existing lists of conditions led to the selection of 60 conditions that can be used when defining multimorbidity for research focused on hospitalised patients. ICD-10 codes were identified for each of these conditions to ensure consistency in their operationalisation.

**Conclusions:**

This work contributes to achieving the goal of greater transparency and consistency in the approach to the study of multimorbidity, with a specific focus on the UK hospital setting.

**Supplementary Information:**

The online version contains supplementary material available at 10.1007/s41999-024-00953-8.

## Key summary points

**Aim:**

To propose a set of principles that can be used to select long-term conditions when studying multimorbidity in hospitalised patients and apply these principles to identify a list of conditions.

**Findings:**

We have outlined a list of principles and applied these to identify a list of 60 long-term conditions that can be utilised when conducting research on multimorbidity in hospitalised patients in the UK and other countries with similar population health profiles.

We have mapped this list of 60 conditions to the International Classification of Diseases 10th revision (ICD-10) codes, drawing on clinical and coding expertise, to facilitate consistency in the operationalisation of this list.

**Message:**

Our work addresses the need for greater transparency and consistency in the approach to the definition of multimorbidity and provides clear recommendations for those conducting research on multimorbidity in the hospital context.

**Supplementary Information:**

The online version contains supplementary material available at 10.1007/s41999-024-00953-8.

## Introduction

The rising global prevalence of multimorbidity, also referred to as multiple long-term conditions, is widely recognised as one of the most important challenges currently facing medicine and population health [1–5]. Many funders of health-related research have responded to this challenge by prioritising work that addresses the need to improve the lives of the growing numbers of people living with multimorbidity. However, progress in this rapidly expanding field of research has been hampered by a lack of transparency and major inconsistencies in methods of operationalising definitions of multimorbidity [6–8].

A high-level definition of multimorbidity—the ‘co-occurrence of two or more long-term conditions’—has been outlined by organisations including the UK Academy of Medical Sciences and the National Institute for Health and Care Excellence [2, 9]. However, a systematic review of studies of multimorbidity [6], found that while this high-level definition has been widely adopted, over a third of 566 studies still did not report this or any other definition of multimorbidity. In addition, major variation was found in the type and number of different conditions researchers had used when operationalising multimorbidity, where this was reported. This is an important source of inconsistency, particularly in epidemiological studies. For example, a recent study of UK primary care data found major variation in the prevalence of multimorbidity (i.e. having two or more long-term conditions) dependent on the number and types of conditions selected [10].

Identifying solutions that ensure greater transparency and consistency in the approach to the selection of conditions being used to define multimorbidity for research across different settings is essential. A recent Delphi consensus study therefore provides a promising way forward for the research community [7, 8]. This study involved consulting over 150 professionals and 25 public participants. Its key outputs were a set of recommendations that can be adapted and applied to identify conditions in different contexts and, a consensus list of 59 conditions that it was recommended should always (*n* = 24) or usually (*n* = 35) be included when defining multimorbidity [7]. A study has shown that utilising this list of 59 conditions may identify more people living with multimorbidity in primary care data than other published lists of conditions supporting its use [10]. However, there is recognition that this list may still need adapting dependent on the research question and setting [7, 8] and one setting where further work is clearly warranted is in secondary care [11].

The ADMISSION research collaborative aims to transform the understanding of multimorbidity in hospital patients. We therefore followed the recommendations outlined by Ho and colleagues [7] to develop a set of principles that could be applied to select a list of conditions relevant to research on multimorbidity in people admitted to hospitals in the UK. Our goal was to produce code lists for each selected condition to ensure full transparency and, consistency in work using this list across different datasets.

## Methods

As a first step, we considered the recommendations on types of conditions to include when defining multimorbidity outlined by Ho and colleagues [7]. Co-authors discussed adaptations required to these recommendations to ensure their relevance to the study of multimorbidity in a hospital setting resulting in the production of an agreed set of principles.

These principles were then applied, independently by two authors who are physicians specialising in geriatric medicine and thus have clinical knowledge of a broad range of conditions relevant to multimorbidity, to two key lists of conditions:

1. the 59 conditions identified via Ho and colleagues’ Delphi consensus study [7],
2. the 100 most prevalent conditions recorded in Hospital Episode Statistics (HES) for England for the date range 1 April 2018 to 31 March 2019 (to avoid the impact of subsequent disruptions to hospital services caused by the COVID-19 pandemic) (see Supplementary table 1) [12].

The first of these lists was selected because its use has been recommended to ensure comparability of multimorbidity definition across studies [7, 10]. The second list was considered to aid identification of additional conditions important in the UK hospital context.

For each of the conditions on the list of 59 conditions, the two clinical reviewers were asked to recommend including them unless there was a compelling reason, when compared against the agreed principles, for their exclusion. Conversely, of the conditions on the list of 100 most prevalent conditions in HES data not also on the 59 conditions list, the clinical reviewers were asked to recommend excluding them unless there was a compelling reason for their inclusion.

After the two clinical reviewers had independently assessed whether each condition should be included or not, their recommendations were combined. A meeting of co-authors, including the two clinical reviewers, two senior clinical academics in geriatric medicine and an epidemiologist, was then convened. At this meeting, any disagreements in recommendations were discussed and resolved. In addition, those conditions from the list of 59 conditions that the reviewers had recommended excluding and those from the list of 100 HES conditions that they had recommended including were carefully considered. The list of conditions resulting from this process was then presented to the ADMISSION Programme Management Group comprising an additional six researchers from a range of clinical specialties and academic disciplines and two public co-investigators for further discussion, adjudication and ratification.

To ensure consistency in the operationalisation of the ratified list of conditions, ICD-10 codes (version 2019) were assigned to each condition. Where possible, lists of codes published on the HDR UK phenotype library [13] were utilised. In the first instance we used those code lists recommended by the CALIBER research platform [14] that are published on the HDR UK phenotype library. Where these were not available other code lists published on the library were used. It should be noted that a decision was taken to use ICD-10 codes rather than ICD-11 codes. This was because ICD-11 codes are not expected to be mandated for use by NHS England until at least 2026 [15] and so have not yet been widely adopted by clinical coding teams in the UK.

After expanding relevant ICD-10 codes to four or five characters where required to maximise capture, the code lists for each condition derived from the HDR UK phenotype library were reviewed by two authors. Based on their clinical assessment, if a specific code did not align with the underlying diagnostic construct described by the condition on our list it was removed. In addition, where required to ensure a more complete capture of relevant diagnoses of a specific condition, additional codes were proposed and agreed upon by the author group. For those conditions not included in the HDR UK phenotype library, an author with clinical expertise generated lists of ICD-10 codes and sought consensus from other authors on the appropriateness and completeness of these. Once ICD-10 codes had been assigned to all conditions on our list these were reviewed by the clinical coding team at the Newcastle Hospitals NHS Foundation Trust. Based on their recommendations, additional ICD-10 codes were added where required to ensure the alignment of our code lists with current standard coding practices within hospitals in England.

UK Biobank is being widely used by the multimorbidity research community, including within ADMISSION [16]. To ensure work we undertake in a hospital setting can be translated to the general population and vice versa, we also identified the relevant variable codes for each condition on our list within the baseline assessment of UK Biobank.

### Patient and public involvement

The ADMISSION research collaborative has two public co-investigators and a Patient Advisory Group (PAG) comprising patients and carers all with lived experience of multimorbidity. The PAG have been involved at every stage of research from the identification of research priorities and development of scientific questions in preparing the funding bid to regular engagement in the research we are now developing and delivering. We work closely with the co-investigators and PAG members and value the advice and guidance they provide on all components of the research programme. This includes the contributions of our two public co-investigators to discussions at the ADMISSION Programme Management Group meeting where the final list of conditions was adjudicated and ratified. We have kept PAG members updated on our progress with this work at 4-monthly meetings and in due course the recommendations from this study will be disseminated to relevant patient and public communities and other stakeholders as part of our comprehensive communications strategy.

## Results

**Fig. 1:**
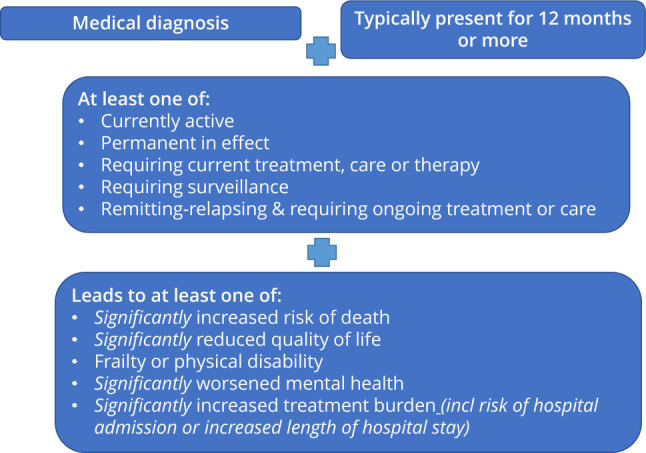
Principles for the selection of conditions for inclusion within the ADMISSION research collaborative’s work on multimorbidity in hospital patients (adapted from Ho and colleagues (2022) [7])

By adapting recommendations outlined by Ho and colleagues [7], we agreed on the following set of principles that would be applied to identify health conditions for inclusion in our work on multimorbidity in hospital patients. As shown in Fig. 1, it was agreed that all conditions must meet the following four criteria:

1. be a medical diagnosis,
2. be typically present for 12 months or more based on our understanding of the usual course of the condition,
3. be at least one of currently active; permanent in effect; requiring current treatment, care or therapy; requiring surveillance; remitting-relapsing and requiring ongoing treatment or care,
4. lead to at least one of significantly increased risk of death; significantly reduced quality of life; frailty or physical disability; significantly worsened mental health; significantly increased treatment burden (indicated by an increased risk of hospital admission or increased length of hospital stay).

When these principles were applied to the list of 59 conditions identified via Ho and colleagues’ Delphi consensus study [7], there was agreement that three conditions on the ‘usually include’ list should be excluded: transient ischaemic attack (TIA), benign cerebral tumours and post-acute COVID-19. By definition TIA resolves within 24 h and so does not meet the criterion of typically being present for 12 months or more, in addition, a related outcome, stroke is included as a separate condition. The decision to exclude benign cerebral tumours was based on the incidental nature of many diagnoses, and their consequently limited impact on prognosis, symptoms and healthcare use in most cases. Post-acute COVID-19 (“Long COVID”) was excluded as this condition currently lacks a stable definition or agreed list of diagnostic features and there were concerns that it would not be consistently coded in different datasets.

Of the 100 most prevalent conditions recorded in HES data in 2018–2019, 33 were also included on the list of 59 conditions. Of the other 67 conditions, it was agreed that there was a compelling reason to include four: diverticular disease, gastro-oesophageal reflux disease, hyperplasia of the prostate and respiratory failure (not otherwise specified). In each case, the conditions were deemed to be common causes of hospital admission, to carry a significant symptom burden and require medical investigation and treatment.

After discussions with the ADMISSION Programme Management Group, it was agreed that ‘chronic urinary tract infection’, from the usually include a list of 59 conditions, should be referred to as ‘recurrent urinary tract infection’, recognising that in most cases, recurrent episodes rather than persistent infection is the underlying pattern of presentation and pathology. In addition, it was agreed that ‘respiratory failure (not otherwise specified)’, selected from the HES list, should be included as the more narrowly, but more clearly defined condition of ‘obstructive sleep apnoea’ to aid its detection in hospital record data; this condition fulfils the other criteria specified above. All other decisions were confirmed and ratified by the Programme Management Group. The final agreed list of 60 conditions is presented in Table 1. Code lists for the operationalisation of these conditions using ICD-10 codes and baseline data from UK Biobank are presented in Supplementary Table 2 and 3, respectively. Of the ICD-10 codes assigned to each of the 60 conditions, 52 were derived using code lists published on the HDR UK phenotype library (27 with no changes, 9 with some codes omitted, 13 with additional codes added and 3 with some codes omitted and other codes added) and 8 by creating our own code lists. In UK Biobank all but five of the 60 conditions had been captured in the baseline assessment.

**Table 1:** Conditions selected for inclusion in research on multimorbidity in hospitalised patients ordered alphabetically by body system

Body system (based on ICD-10 chapters)	Conditions for inclusion (*n* = 60)^a^
Cancer	Haematological cancers
	Melanoma
	Metastatic cancers
	Solid organ cancers
Cardiovascular disease	Aneurysm
	Arrhythmia
	Coronary artery disease
	Heart failure
	Heart valve disorders
	Hypertension
	Peripheral artery disease
	Stroke
	Venous thromboembolic disease
Congenital disease	Congenital disease and chromosomal abnormalities
Digestive disease	Chronic liver disease
	Chronic pancreatic disease
	Diverticular disease^b^
	Gastro-oesophageal reflux disease^b^
	Inflammatory bowel disease
	Peptic ulcer
Ear disease	Hearing impairment that cannot be corrected
	Ménière’s disease
Eye disease	Vision impairment that cannot be corrected
Haematological disorder	Anaemia (including pernicious anaemia, sickle cell anaemia)
Infectious disease	Chronic Lyme Disease
	HIV/AIDS
	Tuberculosis
Mental and behavioural disorder	Anxiety
	Autism
	Bipolar disorder
	Dementia
	Depression
	Drug or alcohol misuse
	Eating disorder
	Post-traumatic stress disorder
	Schizophrenia
Metabolic and endocrine disease	Addison’s disease
	Cystic fibrosis
	Diabetes mellitus
	Thyroid disorders
Musculoskeletal disease	Connective tissue disease
	Gout
	Long-term musculoskeletal problems due to injury
	Osteoarthritis
	Osteoporosis
Neurological disease	Chronic primary pain
	Epilepsy
	Multiple sclerosis
	Paralysis
	Parkinson’s disease
	Peripheral neuropathy
Respiratory disease	Asthma
	Bronchiectasis
	Chronic obstructive pulmonary disease
	Obstructive sleep apnoea^b, c^
Urogenital disorder	Chronic kidney disease
	Endometriosis
	End-stage kidney disease
	Hyperplasia of the prostate^b^
	Recurrent urinary tract infection^d^

*ICD-10* International classification of diseases, 10th revision

^a^ Selected by applying principles outlined in Fig. 1 to a list of 59 conditions proposed by Ho and colleagues (2022)[7] unless otherwise specified

^b^ Selected by applying principles outlined in Fig. 1 to a list of the 100 most prevalent conditions recorded in diagnosis data from hospitals in England in 2018–2019[12]

^**c**^ Included to identify people coded as’respiratory failure (not otherwise specified)’

^d^ Replaces ‘chronic urinary tract infection’ as chronicity will be determined in hospital records by assessing recurrence

## Discussion

In this paper, we have outlined the structured and transparent approach taken to adapt and then apply a set of principles that have resulted in the selection of a list of 60 long-term conditions that we recommend are included in research studies of multimorbidity in hospitalised patients. This underpins our work to understand the mechanisms, causes and consequences of multimorbidity in hospitalised patients in the UK. It ensures our definition of multimorbidity is always explicitly defined, clinically meaningful and applicable to a hospital setting and, that conditions included and principles for their selection are clearly reported.

A key strength of this work is its alignment with the recommendations made in a recent Delphi consensus study [7]. This was an active decision taken to ensure comparability with work that it has been predicted will soon be the reference standard for the multimorbidity definition [8]. While we could have drawn on any one of a number of other published lists of conditions [14, 17–20] including the list used in other research undertaken in a hospital setting in the UK [20, 21], we chose to work with the list of long-term conditions produced via the Delphi consensus study by Ho and colleagues [7] for a number of reasons. First, not only did Ho and colleagues produce a recommended list of conditions but they first provided a set of clear recommendations for the selection of conditions which we were able to adapt to create our own principles relevant to the study of multimorbidity in the hospital context. Second, the consensus study drew on a large panel of international experts alongside a smaller panel of members of the public some of whom had lived experience of multimorbidity. Third, recent evidence has shown that the list proposed by Ho and colleagues identifies more people living with multimorbidity in primary care data than many other published lists of conditions. By using this list, researchers will be close to a ceiling effect above which adding more conditions is unlikely to meaningfully impact on prevalence estimates of multimorbidity [10]. This is an important consideration given the need to balance the benefits of increasing the number of conditions against the practicality of capturing all these conditions reliably in different types of dataset (including primary and secondary care electronic health records and population-based studies).

When our list of 60 conditions was compared with other published lists there was often considerable overlap [17, 18, 20] except where the other list was much more extensive and included many health conditions which are arguably not long-term [14]. This provides further reassurance that our approach will enable comparability not only within the different work streams of our own research collaborative but also across studies and research teams.

Another key strength of our work is the identification and provision of ICD-10 codes for each condition on our list. This should enable consistency in the operationalisation of the list thus removing another potentially important source of heterogeneity between studies—variation in the codes used to identify specific conditions in different datasets and by different research teams. However, while we have drawn on published lists of ICD-10 codes for our conditions [13, 14] where available, in a small number of cases these did not exist and so we have created our own coding lists based on clinical expertise within the authorship team and consultation with members of a clinical coding team at the Newcastle Hospitals NHS Foundation Trust. While we consulted a clinical coding team to try and ensure the alignment of our proposed list of ICD-10 codes with current standard coding practices within hospitals in England, this assumes coding practices are similar in different hospital trusts which may not be the case. We therefore expect that further work will be required to refine the code lists, not least because coding teams will be transitioning to the use of ICD-11 codes in the future [15]. However, our multi-stage review process of code lists did focus on maximising the capture of all listed conditions to allow for different coding practices within different organisations.

There are other challenges faced in achieving the full capture of all listed conditions including the fact that some conditions on the list are not easily defined (for example, paralysis) and that not all conditions are well captured in electronic health records [22–25] especially when using ICD-10 codes alone. In due course, we, therefore, plan to consider how best to augment ICD-10 code data with other routinely captured structured data such as laboratory results and prescribing data and to use natural language processing to extract relevant information from unstructured data such as medical notes [26, 27].

Two co-authors with clinical expertise independently applied the agreed principles for the selection of conditions to two existing lists of conditions prior to their decisions being compiled and discussed at a meeting of the wider co-authorship group. Although independent assessments by co-authors able to draw on their clinical expertise is another strength of our work, we recognise that the judgements made have an element of subjectivity and a different team of authors may have made a different set of decisions. However, where there was any outstanding uncertainty about specific conditions after discussion among the co-author group, there was an opportunity to discuss these with the ADMISSION Programme Management Group prior to the final list being ratified. In addition, the decisions taken and the reasons for these are clearly documented. This highlights the need to recognise that no one list of conditions will ever be definitive and that researchers may need to adapt the list they use to meet their specific requirements. Most important is to be transparent about the principles applied, the decisions taken and the list utilised—even if these differ from other research groups, their reporting will aid comparison and help move the field of multimorbidity research forward.

Due to the transparent approach taken, the list of conditions we provide is likely to have utility beyond the UK and can be adopted by researchers investigating multimorbidity in hospital settings in countries with similar population health profiles to the UK. However, we acknowledge that the list will not be applicable in all countries, including many low- and middle-income countries where there are differences in the distribution of long-term conditions including a higher prevalence of some long-term conditions not commonly observed in the UK. When studying multimorbidity in countries with markedly different population health profiles to the UK, we would advise researchers to apply the principles we have outlined to identify a list of conditions relevant to their specific context.

Our primary focus in this paper was defining multimorbidity for research purposes. Establishing whether the list we have derived could also be used in clinical practice is beyond the scope of this paper, but further research is warranted to assess this.

In the rapidly expanding field of multimorbidity research it is widely acknowledged that methodological advancements are required to improve transparency and consistency of approach to the definition of multimorbidity. This will ensure that future studies of multimorbidity are more generalisable and comparable. We have proposed an adapted set of principles that can be used to select long-term conditions when studying multimorbidity in hospitalised patients. We have then applied these principles to identify a list of 60 long-term conditions and provided guidance on how these can be operationalised. Our work, which we hope will be adopted by others, contributes to the goal of achieving greater transparency and consistency in the approach to research on multimorbidity in the hospital setting. The ultimate aim of this is to improve the lives and care experiences of the growing number of people living with multimorbidity.

## Supplementary Information

Below is the link to the electronic supplementary material.

Supplementary file1 (XLSX 117 kb)
